# Construction and Analyses of Human Large-Scale Tissue Specific Networks

**DOI:** 10.1371/journal.pone.0115074

**Published:** 2014-12-16

**Authors:** Wei Liu, Jianying Wang, Tengjiao Wang, Hongwei Xie

**Affiliations:** 1 College of Mechanical & Electronic Engineering and Automation, National University of Defense Technology, Changsha, China; 2 The Affiliated Hospital of Military Medical Sciences, Beijing, China; Texas A&M University, United States of America

## Abstract

Construction and analyses of tissue specific networks is crucial to unveil the function and organizational structure of biological systems. As a direct method to detect protein dynamics, human proteome-wide expression data provide an valuable resource to investigate the tissue specificity of proteins and interactions. By integrating protein expression data with large-scale interaction network, we constructed 30 tissue/cell specific networks in human and analyzed their properties and functions. Rather than the tissue specificity of proteins, we mainly focused on the tissue specificity of interactions to distill tissue specific networks. Through comparing our tissue specific networks with those inferred from gene expression data, we found our networks have larger scales and higher reliability. Furthermore, we investigated the similar extent of multiple tissue specific networks, which proved that tissues with similar functions tend to contain more common interactions. Finally, we found that the tissue specific networks differed from the static network in multiple topological properties. The proteins in tissue specific networks are interacting looser and the hubs play more important roles than those in the static network.

## Introduction

A static protein-protein interaction (PPI) network describes a set of physical associations that can occur between proteins. However, only a subset of proteins can be expressed and interact with each other in any particular cell or tissue. Integrating interaction and expression data, we can analyze the interplay between protein expression and physical interactions in humans. The basic idea is taking the static protein interaction network as a skeleton, and searching specific sub-networks from it, according to the expression level changes of proteins in different tissues. Its main goal is constructing tissue specific PPI networks in order to understand the dynamics of biological systems [1]–[3].

Through analyzing genome-wide gene expression patterns, researchers proposed plenty of methods to identify tissue specific and ubiquitously expressed (housekeeping) genes [4], [5]. For example, Dezso et al. measured whole genome expression in 31 human tissues, identifying 2,374 housekeeping genes expressed in all tissues, and genes uniquely expressed in each tissue [6]. Bossi et al. combined a large-scale protein interaction network with gene expression profiles in 79 tissues, to identify tissue specific proteins [7]. They found that the tissue specific proteins have fewer physical interactions and tend to be recently evolved proteins, compared with the universally expressed proteins. Most tissue specific proteins do, however, bind to universally expressed proteins. This result was further confirmed by Zhu et al.'s work [8]. These investigations showed that there were significant differences between housekeeping proteins and tissue-specific proteins, in both network properties and functions.

Similar to the tissue specificity of genes, the tissue specificity of interactions were also investigated [9], [10]. Lopes et al. combined the PPI network from multiple databases with gene expression data from 84 tissues/cells, and constructed tissue specific sub-networks [9]. They found that tissue specific sub-networks possess significantly fewer interactions than the original PPI databases (between 1∼25%). These sub-networks are considerably more fragmented than the parent networks, but they have stronger biological relevance with the tissues and more high-confidence interactions. In addition, the comparison of tissue specific sub-networks with the global static network is of great importance to establish high-confidence interaction networks.

However, these findings were all based on the tissue specific networks inferred from gene expression data. With the announcement of the human proteome map [11], [12], it is necessary to construct large-scale tissue specific networks based on protein expression data and comprehensively analyze their functions and network properties. In this paper, we firstly identified the tissue specific proteins and housekeeping proteins and analyzed their particular interacting patterns. Secondly, we focused on the tissue specificity of interactions to establish tissue specific PPI networks. Thirdly, we investigated the differences between tissue specific networks inferred from gene expression data and protein expression data, as well as the similarity of various tissue specific networks. Finally, we computed the topological properties of tissue specific networks and the static network in order to reveal the structure characteristic of tissue specific networks.

## Materials and Methods

### Human protein expression data

Human protein expression data is coming from the dataset reported by Kim et al., who presented a draft map of the human proteome using high-resolution Fourier-transform mass spectrometry [11]. This dataset contained the proteomic profiling of 30 histologically normal human samples, including 17 adult tissues, 7 fetal tissues and 6 purified primary haematopoietic cells, resulted in identification of proteins encoded by 17,294 genes accounting for approximately 84% of the total annotated protein-coding genes in humans.

### Conservation analysis

We calculated the dN/dS values for all expressed proteins to characterize their evolution rates (S1 Table). The synonymous and non-synonymous substitution rates between human and mouse were obtained from Ensembl (http://www.ensembl.org/biomart/martview/).

### The integrated human protein interaction network

We established an integrated human protein interaction network by combing the PPI data from multiple databases. Firstly, we downloaded the global human physical protein interaction network from previous material [7], including data from 21 different sources to form a network of 80,922 physical interactions between 10,229 human proteins. To enlarge the scale of protein interaction data, we then combined it with the iRefIndex database [13], which extracted original PPIs from BIND, BioGRID, CORUM, DIP, HPRD, IntAct, MINT, MPact, MPPI and OPHID database. To ensure the reliability of protein interactions, only interactions supported by at least one piece of direct experimental evidence were included to demonstrate physical association between two human proteins. Through unifying protein accessions and deleting redundant interactions, we finally established a PPI network, containing 18,425 proteins and 193,273 interactions (S2 Table).

## Results

### Identification of tissue specific proteins and interactions

We defined a protein to be universal if it was expressed in all the 30 tissues and cell lines. Accordingly, tissue specific proteins are the proteins expressed in only one tissue or cell line. Based on human protein expression data of Kim et al. [11], we found that proteins expressed in more tissues/cells tend to have higher expression level (Pearson correlation coefficient R = 0.22, P = 7.00×10^−192^) and more interacting neighbors (R = 0.20, P = 1.32×10^−124^). At the same time, proteins expressed in more tissues/cells tend to have lower evolutionary rate (R = −0.23, P = 1.96×10^−180^), in accordance with the previous result obtained from mRNA dataset [7]. The method to obtain the evolutionary rates of proteins was given in the section of Materials and Methods. Then, we defined an interaction as tissue specific interaction if their interacting proteins can be co-expressed in one tissue/cell, or housekeeping interaction if their interacting proteins co-expressed in all tissues/cells. In theory, only if two genes express simultaneously in a tissue/cell, their products can interact with each other in some specific conditions. Based on protein expression data of Kim et al. [11], we identified 6,792 tissue specific interactions and 22,069 housekeeping interactions (S3 Table).

**Figure 1:**
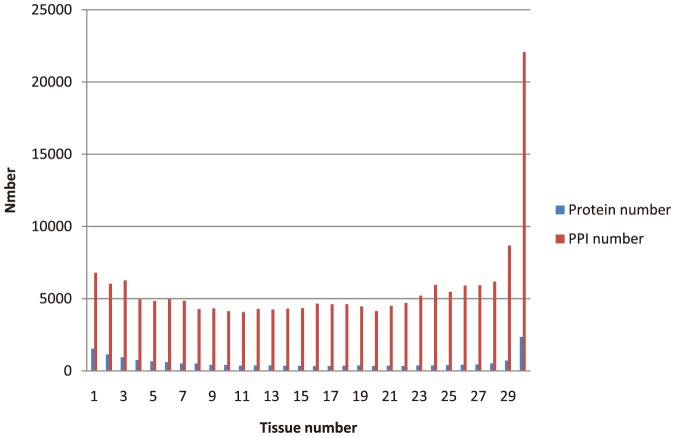
The distribution of proteins and interactions expressed in tissues/cells.

The number of tissue/cell types where a protein/interaction was observed was counted, as shown in Fig. 1. There are certain differences between the expression distribution of proteins and interactions in tissues/cells. The distribution of proteins has a obvious trough of wave, meaning that the number of universally expressed and selectively expressed proteins are much larger than those of proteins expressed in 2∼29 tissues/cells. However, the distribution of interactions followed an unimodal pattern, in which the number of housekeeping interactions is much larger than those of proteins expressed in 1∼29 tissues/cells. This result inferred from the dataset of Kim et al. [11] indicated that the universally expressed proteins tend to interact with other universally expressed proteins, while selectively expressed proteins tend to interact with all kinds of proteins.

**Figure 2:**
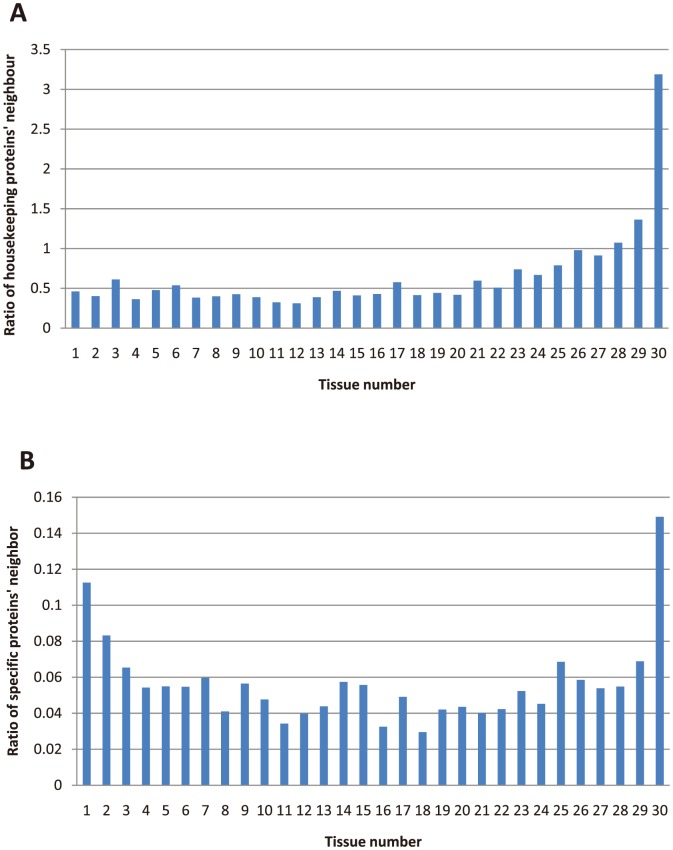
The interaction ratio of (A) housekeeping proteins and (B) tissue specific proteins.

To avoid the bias of single protein expression dataset, we used the overlap of proteins from the datasets of Kim et al. [11] and Wilhelm et al. [12] to determine tissue specific proteins and housekeeping proteins. As a result, we found 627 tissue specific proteins and 1,093 housekeeping proteins (S4 Table), which were proteins expressed in one tissue/cell or all tissues/cells in both protein expression datasets. To illustrate the interacting pattern of proteins, we counted the number of proteins interacting with housekeeping proteins and tissue specific proteins according to their different tissue expression number. To eliminate the influence of tissue expression number, we computed the average interaction ratio of proteins according to their tissue expression number (Fig. 2). It can be confirmed that housekeeping proteins and tissue specific proteins have significantly different interacting patterns, meaning that compared with housekeeping proteins, tissue specific proteins are more likely to interact with all kinds of proteins. Especially, tissue specific proteins have extensive interactions with housekeeping proteins, which are usually core cellular components in the PPI network. This trend well coincides with the previous result obtained from mRNA dataset [7].

### Construction of tissue specific networks

We used human protein expression data to determine the tissues/cells of the human body in which protein interactions can occur. If two proteins co-expressed in a tissue/cell and meanwhile they are available in the integrated human protein interaction network, then they can physically interact with each other in this tissue/cell in some conditions. By identifying all the specific interactions in each tissue/cell, we established the tissue specific networks in 30 tissues/cells (S5 Table). Differed from the static network with 13,509 proteins and 172,848 interactions, only a part of proteins (88.05∼91.55%) and interactions (50.06∼62.95%) were observed in the tissue specific networks. As shown in Table 1, the percent of interactions in tissue specific networks occupying the static network is generally lower than that of proteins. This result is reasonable because the tissue specific interaction required the co-expression of two proteins, which is more difficult to achieve than the expression of single protein. Since proteins usually play roles by interacting with each other, we deemed that it is more meaningful to analyze the tissue specific interactions rather than tissue specific proteins. In addition, the tissue specific networks established by our methods are much larger than those reported by Lopes et al. [9]. The reason may be lie in the high coverage of human protein expression data we used.

**Table 1:** Tissue specific networks inferred from protein expression data.

Tissue/Cell	Number of proteins	Percent of proteins(%)	Number of interactions	Percent of interactions(%)
Fetal Heart	12,368	91.55	101,864	58.93
Fetal Liver	12,055	89.24	92,323	53.41
Fetal Gut	12,522	92.69	108,548	62.80
Fetal Ovary	12,096	89.54	93,672	54.19
Fetal Testis	12,365	91.53	104,401	60.40
Fetal Brain	11,972	88.62	86,524	50.06
Adult Frontal Cortex	12,374	91.60	101,715	58.85
Adult Spinal Cord	12,081	89.43	92,704	53.63
Adult Retina	12,506	92.58	108,809	62.95
Adult Heart	12,151	89.95	93,794	54.26
Adult Liver	12,391	91.72	104,517	60.47
Adult Ovary	11,962	88.55	86,563	50.08
Adult Testis	12,376	91.61	101,865	58.93
Adult Lung	12,106	89.61	92,324	53.41
Adult Adrenal	12,506	92.58	108,549	62.80
Adult Gallbladder	12,157	89.99	93,673	54.19
Adult Pancreas	12,389	91.71	104,402	60.40
Adult Kidney	11,965	88.57	86,525	50.06
Adult Esophagus	12,361	91.50	101,716	58.85
Adult Colon	12,112	89.66	92,705	53.63
Adult Rectum	12,551	92.91	108,810	62.95
Adult Urinary Bladder	12,138	89.85	93,795	54.26
Adult Prostate	12,381	91.65	104,518	60.47
Placenta	11,894	88.05	86,564	50.08
B Cells	12,396	91.76	101,865	58.93
CD4 Cells	12,109	89.64	92,325	53.41
CD8 Cells	12,534	92.78	108,550	62.80
NK Cells	12,162	90.03	93,674	54.19
Monocytes	12,379	91.64	104,403	60.40
Platelets	11,931	88.32	86,525	50.06
Static network	13,509	100	172,848	100

### Comparison of tissue specific networks inferred from protein and mRNA expression data

**Figure 3:**
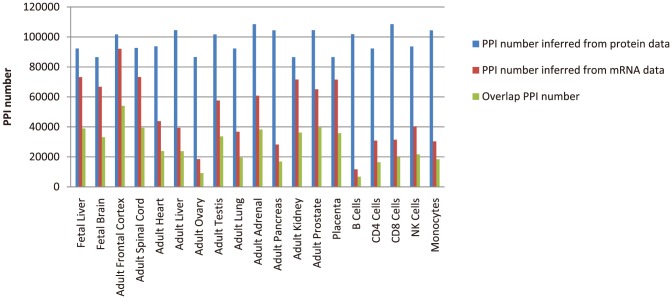
The comparison of tissue specific networks inferred from protein and mRNA expression data.

Investigators usually identified tissue specific networks from gene expression data, before the proteome-wide protein expression data was available. For typically, Bossi et al. used gene expression data [14] to determine the cells and tissues of the human body in which each of these interactions can occur [7]. If two genes are co-expressed in a cell, then under some conditions their products can physically interact in that cell. To compare these two different data sources, we extracted tissue specific networks in 19 tissues/cells inferred from human protein and mRNA expression data (Fig. 3).

As shown in Fig. 3, the scales of most tissue specific networks inferred from protein expression data are larger than those of networks inferred from mRNA expression data. Especially, the overlap of interactions inferred from different data sources only occupied about a half of those inferred from protein expression data (10.58∼53.14%). This can be attributed to that the co-expression genes will not certainly lead to protein interactions. Since mRNA expression data is an indirect method to observe protein expression, we can deem that the tissue specific networks inferred from protein expression data are more credible than those inferred from mRNA expression data. This also means that previous biological findings based on microarrays might have to be re-examined using protein expression datasets.

### Analyses of overlap interactions across tissue specific networks

**Figure 4:**
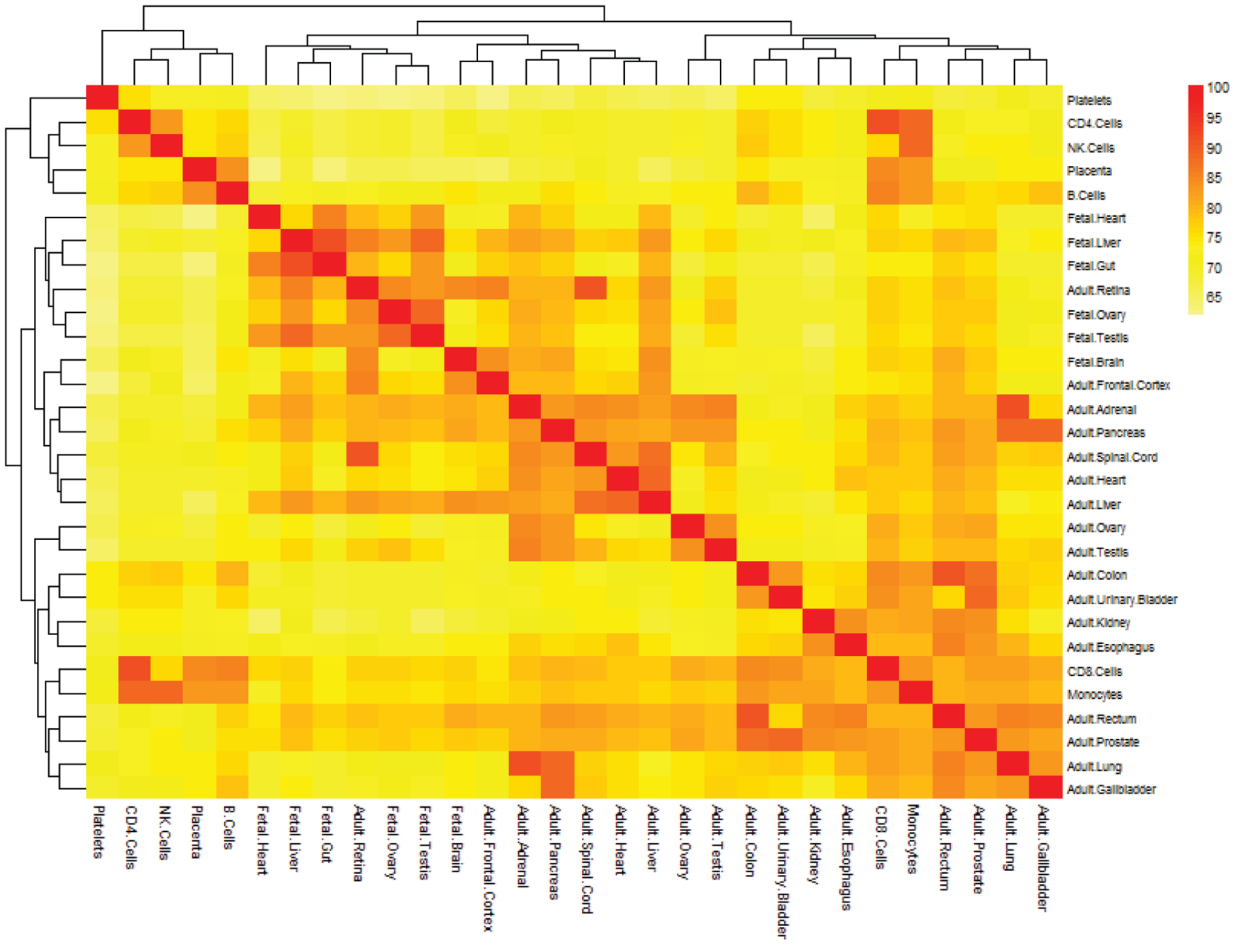
The heat map of network similarity.

Based on the tissue specific networks established, we counted the number of common interactions across different tissue specific networks. By computing the percent of overlap interactions occupying each tissue specific network, the similarity of different networks can be investigated (Fig. 4). Some tissue specific networks show very high similarity, such as fetal liver and fetal gut (90.88%), adult retina and adult spinal cord (90.65%), adult colon and adult rectum (90.65%). In general, we can infer that tissues with similar functions tend to contain more common interactions. By contrast, there are only moderate similarity between fetal tissues and their corresponding adult tissues, for example fetal liver and adult liver (83.05%), fetal heart and adult heart (71.08%), indicating the tissue specific networks change largely in the course of growth and differentiation.

**Figure 5:**
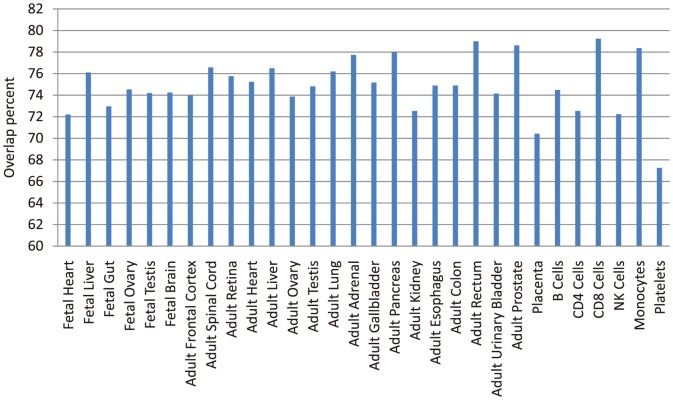
Average overlap percent across different networks occupying each tissue specific network.

By computing the average percent of overlap interactions occupying each tissue specific network, we obtained the extent of tissue specificity in each tissue/cell type (Fig. 5). The result shows that the CD8 Cells have the lowest tissue specificity in all the tissues/cells, because their network has average maximum common interactions (79.23%) with the networks in other tissues/cells. Platelets have the strongest tissue specificity, since their tissue specific network has average fewest common interactions (67.26%) with other networks. In general, there is a relatively high percent of common interactions across different tissues/cells (average 67.26∼79.23%), indicating different tissues/cells usually play roles through similar interactions or working mechanism despite of their various functions.

### Analyses of topological properties in tissue specific networks

As reported, most biological networks have scale-free, small-world property and modularity [15]. Here, we investigated the general topological properties of tissue specific networks, in order to reveal their particular characteristics compared with the static PPI network.

We selected six typical topological indexes of networks, including degree exponent, average degree, average path length, diameter, betweeness and average clustering coefficient. Based on the degree of individual nodes, the degree distribution of a network, P(k), is defined, which gives the probability that a selected node has exactly k links. Most biological networks are scale-free [16], which means that their degree distribution approximates a power law, P(k)∼k^−γ^, where γ is the degree exponent. The smaller the value of γ, the more important the role of the hubs is in the network. An undirected network with N nodes and L links is characterized by an average degree <k> = 2L/N. The mean path length represents the average over the shortest paths between all pairs of nodes and offers a measure of a network's overall navigability [17]. The diameter of a network is the longest shortest path within a network. Betweeness of network represents the average loading flux of all nodes. In addition, the average clustering coefficient characterizes the overall tendency of nodes to form clusters or groups [18]. The closer the local clustering coefficient is to 1, the more likely it is for the network to form clusters.

**Figure 6:**
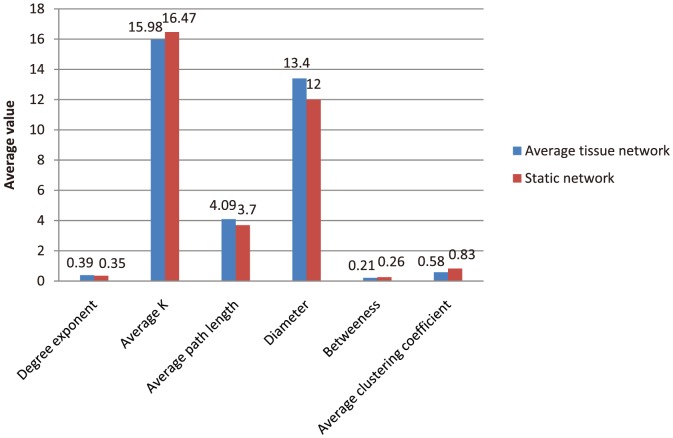
Average topological indexes of tissue specific networks and the static network.

Using the network analysis tool Pajek [19] and self-developed programs, we computed six topological indexes of 30 tissue-specific networks and the static network (S6 Table). As most biological networks, the tissue specific networks have scale-free, small-world property and modularity. However, the tissue specific networks have larger degree exponents (P = 8.96×10^−29^), network diameter (P = 7.35×10^−10^) and mean path lengths (P = 3.02×10^−36^), smaller average degrees (P = 0.013), betweeness (P = 1.49×10^−34^) and average clustering coefficients (P = 1.69×10^−27^) than the static network (Fig. 6). This result indicated that the tissue specific networks are linked looser, their hubs play more important roles, they have longer communication paths and contain less clusters than the static network.

## Conclusion

In this paper, we constructed 30 tissue specific networks based on human protein expression data, and analyzed their properties and functions. Through the analyses of tissue specific networks, we found that housekeeping proteins and tissue specific proteins have significantly different interacting patterns. The housekeeping proteins tend to interact with other housekeeping proteins, while tissue specific proteins tend to interact with all kinds of proteins. Especially, we focused on the tissue specificity of interactions considering which is higher than that of genes. Due to the high coverage of protein expression data, we established larger-scale tissue specific networks than those reported in [9]. Based on the overlap interactions across different tissues/cells, we compared the similar extent of multiple tissue specific networks. As a result, we found that tissues with similar functions tend to contain more common interactions, while the tissue specific networks change largely in the course of growth and differentiation. Furthermore, we found that the tissue specific networks differed from the static network in many aspects. The proteins in tissue specific networks are interacting looser and have less interacting neighbors than those in the static network. These findings can help understand the function and structure of tissue specific networks and reveal the inherent working mechanism of biological systems.

## Supporting Information

S1 Table **The integrated human protein interaction network.**
(XLSX)

S2 Table **Evolutionary rates of expressed proteins.**
(XLSX)

S3 Table **Housekeeping and tissue specific interactions.**
(XLSX)

S4 Table **Housekeeping and tissue specific proteins inferred from two protein expression datasets.**
(XLSX)

S5 Table **Tissue specific PPI networks in 30 tissues/cells.**
(XLSX)

S6 Table **Topological indexes of 30 tissue specific networks and the static network.**
(XLSX)
